# Standard Sub-Thermoneutral Caging Temperature Influences Radiosensitivity of Hematopoietic Stem and Progenitor Cells

**DOI:** 10.1371/journal.pone.0120078

**Published:** 2015-03-20

**Authors:** Benjamin J. Povinelli, Kathleen M. Kokolus, Jason W.-L. Eng, Christopher W. Dougher, Leslie Curtin, Maegan L. Capitano, Christi T. Sailsbury-Ruf, Elizabeth A. Repasky, Michael J. Nemeth

**Affiliations:** 1 Department of Molecular and Cellular Biology, Roswell Park Cancer Institute, Elm and Carlton Streets, Buffalo, New York 14263, United States of America; 2 Department of Immunology, Roswell Park Cancer Institute, Elm and Carlton Streets, Buffalo, New York 14263, United States of America; 3 Department of Laboratory Animal Resources, Roswell Park Cancer Institute, Elm and Carlton Streets, Buffalo, New York 14263, United States of America; 4 Department of Medicine, Roswell Park Cancer Institute, Elm and Carlton Streets, Buffalo, New York 14263, United States of America; French Blood Institute, FRANCE

## Abstract

The production of new blood cells relies on a hierarchical network of hematopoietic stem and progenitor cells (HSPCs). To maintain lifelong hematopoiesis, HSPCs must be protected from ionizing radiation or other cytotoxic agents. For many years, murine models have been a valuable source of information regarding factors that either enhance or reduce the survival of HSPCs after exposure of marrow to ionizing radiation. In a recent series of studies, however, it has become clear that housing-related factors such as the cool room temperature required for laboratory mice can exert a surprising influence on the outcome of experiments. Here we report that the mild, but chronic cold-stress endured by mice housed under these conditions exerts a protective effect on HSPCs after both non-lethal and lethal doses of total body irradiation (TBI). Alleviation of this cold-stress by housing mice at a thermoneutral temperature (30°C) resulted in significantly greater baseline radiosensitivity to a lethal dose of TBI with more HSPCs from mice housed at thermoneutral temperature undergoing apoptosis following non-lethal TBI. Cold-stressed mice have elevated levels of norepinephrine, a key molecule of the sympathetic nervous system that binds to β-adrenergic receptors. We show that blocking this signaling pathway *in vivo* through use of the β-blocker propanolol completely mitigates the protective effect of cold-stress on HSPC apoptosis. Collectively this study demonstrates that chronic stress endured by the standard housing conditions of laboratory mice increases the resistance of HSPCs to TBI-induced apoptosis through a mechanism that depends upon β-adrenergic signaling. Since β-blockers are commonly prescribed to a wide variety of patients, this information could be important when predicting the clinical impact of HSPC sensitivity to TBI.

## Introduction

Thermoregulation is an important, evolutionarily conserved autonomic process that has far reaching effects on many other systems and processes. Ambient temperature is a major variable that determines the behavior and the amount of heat-generating metabolism individuals require to maintain core body temperatures. Thus, long-term exposure to even mildly cool (sub-thermoneutral) temperatures has a profound effect on physiology. Altering thermoregulation directly impacts metabolism [1–6]. Excess thermoregulation required in animals housed at cool temperatures affects growth and development [7–12]. Sleep patterns are affected [13–17]. Hormone production and the immune response are also altered [18–24]. Ambient temperature regulates heat food consumption [18,24–27]. Because of their large surface area to volume ratio, facilitating heat loss or gain, mice and other rodents are especially sensitive to their ambient temperature [28]. Despite the large physiological effects, mice are able to maintain a normal body temperature despite sub-optimal environmental temperatures. However, we have recently demonstrated that even mild chronic cold-stress can significantly depress murine immune cell function and anti-tumor immunity [23,29].

Hematopoietic stem and progenitor cells (HSPCs) utilize a complex network of mechanisms to protect themselves from cytotoxic and genotoxic damage. These cells are responsible for maintaining life-long hematopoiesis, which mandates preservation of HSPC function in order to continuously generate mature blood cells[30]. Healthy hematopoiesis is severely impacted by exposure to radiation which damages both HSPCs and mature blood cells and the destruction of hematopoietic capacity is a leading symptom of acute radiation syndrome[31,32]. Low doses of radiation cause anemia and pancytopenia while higher doses of radiation can induce apoptosis, senescence and promote chromosomal translocations in HSPCs [33–35]. Developing effective countermeasures to acute radiation exposure to protect HSPCs requires a full understanding of all factors responsible for promoting radioprotection of HSPCs.

Classic experiments showed that lowering the body temperature of mice or rats to hypothermic levels prolonged survival and increased the LD_50_ of radiation, but little or no information exists on the impact of mild, physiological cold-stress which is sufficient to stimulate additional thermogenesis, but does not result in an actual decline in core body temperature [36,37]. Interestingly, hypothermia has also been linked to the evolutionarily conserved behavior of hibernation during which body temperature, and metabolic rate, are decreased resulting in a markedly delayed response to radiation[38]. In this study, we tested whether chronic mild cold-stress that is standard in mouse colonies used for research, but is not reducing actual body temperature, is influencing HSPC sensitivity to ionizing radiation. Using specially designed housing units to maintain animals at temperatures resulting in mild cold-stress or at thermoneutrality, the ambient temperature which significantly reduces the requirement for increased thermogenesis, we observed that mild cold-stress is indeed prolonging survival after total body irradiation (TBI) and reducing apoptosis of HSPCs. We also observed that the radio-protective effects of mild cold-stress are mediated through the β-adrenergic signaling pathway. Together, these data provide evidence for a previously unrecognized protective mechanism of mild cold-stress that inhibits apoptosis of HSPCs after cytotoxic injury and further shows that underappreciated physiological factors are capable of influencing baseline responses of marrow cells to cytotoxic injuries.

## Materials and Methods

### Mice

Female, 8–10 week old andC57BL/6NCr (C57BL/6) mice were purchased from the National Cancer Institute (Bethesda, MD USA) and maintained under specific pathogen free conditions following a protocol approved for this study by the Institutional Animal Use and Care Committee at Roswell Park Cancer Institute (Protocol Number 757M). All mice were housed at either 22°C (Standard Temperature: ST) or 30°C (Thermoneutral Temperature: TT) in Precision Refrigerated Plant-Growth Incubators (Thermo Fischer Scientific, Waltham, MA USA) with a 12 hour light/dark cycle. Mice were acclimated to this temperature for at least two weeks prior to experiments and they remained at their assigned temperature for the remainder of every experiment. Humidity within incubators was controlled using Top Fin Air Pump AIR 1000 and Top Fin airline tubing. Mice were provided water and Tekland Global 18% Protein Diet (Harlan, Madison, WI) *ad lithium*. Mice were housed 5 or less per cage and Enrich-o’Cobs Premium Blended Enrichment Bedding was provided (The Andersons, Maumee, Ohio). Prior to experimentation mice were acclimated to ST or TT for at least 2 weeks. For radiation experiments, mice received 300, 600 or 900 cGy total-body irradiation from a ^137^Cs source. Mice were monitored daily for signs of morbidity including body weight loss (greater than 20% of starting weight), hunching, lethargy, anorexia, or failure to thrive. Any mice becoming moribund during the course of the experiment were humanely euthanized using CO_2_ inhalation followed by a secondary method of cervical dislocation. At the end of experiments, all remaining mice were humanely euthanized using CO_2_ inhalation followed by a secondary method of cervical dislocation. For specific experiments, mice also received 10 mg/kg propranolol (Sigma Aldrich, St. Louis, MO USA) delivered via intraperitoneal injection for three days.

### Complete Blood Counts

Whole blood was drawn via the saphenous vein or retro-orbital collection using EDTA anti-coagulant. When blood was collected via retro-orbital bleeding mice were anesthetized with 3–5% Isoflurane. Sedation was assessed by compressing the toes before collection commenced. Samples were analyzed for WBCs and hemoglobin using a Hemogen HC5 (Melet Schloesing, Osny, France).

### Norepinephrine Quantitation

Norepinephrine enzyme-linked immunosorbent assays (ELISA) were performed according to the manufacturer instructions (Rocky Mountain Diagnostics, Colorado, CO USA). Plasma was collected using EDTA anti-coagulant. ELISAs were read with a Bio-Tek HT plate reader (Bio-Tek, Winooski, VT USA). Standard curve analysis was performed with GraphPadv6.0a software (GraphPad Software, Inc., La Jolla, CA USA) using a four-parameter logistic curve.

### Flow Cytometry

Bone marrow cells were analyzed using flow cytometry as previously described using a BD LSR II Flow Cytometer[39]. Briefly, cells were stained with APC-conjugated anti-mouse lineage markers (CD4 (clone GK1.5), CD8a (53-6.7), B220 (RA3-6B2), Gr-1 (RB6-8C5), CD11b (M1/70), Ter119 (Ter-119), PE-Cy7-conjugated anti-Sca-1 (D7) and APC-eFluor780-conjugated anti-c-kit (2B8). All antibodies were from eBioscience (San Diego, CA, USA).

#### Apoptosis

For determination of apoptosis, bone marrow cells were stained using the Annexin V-FITC kit (eBioscience) and 4',6-diamidino-2-phenylindole (DAPI) or for active caspase-3 using the FITC Active Caspase-3 Apoptosis Kit (BD Biosciences, San Jose, CA, USA) according to the manufacturer’s instructions.

#### Cell Cycle

Bone marrow cells were extracted in the morning and analyzed for cell cycle status as described [39]. Cell cycle phases were determined by staining cells with Hoechst 33343 (Sigma Aldrich) and APC-conjugated anti-Ki-67 (SolA15,eBioscience). For these experiments, FITC-conjugated anti-mouse lineage marker antibodies were used.

#### Detection of b-Adrenergic Receptor

Bone marrow cells were stained as described prior to fixation with 2% methanol-free paraformaldehyde and permeabilization with 0.5% saponin and 3% FBS in PBS buffer. After permeabilization, cells were stained with rabbit polyclonal anti-β2 adrenergic receptor antibody (Thermo Scientific, PA5-19649) followed by PE-conjugated goat anti-rabbit IgG.

### *In vitro* Experiments

LSK cells were sorted from mice housed under standard conditions as previously described [39]. Cells were cultured in serum free media with 20 ng/ml stem cell factor and thrombopoietin and treated with 1 μM propranolol (Sigma) or isoproterenol (Sigma) for 48 hours prior to irradiation with 300 cGy. Apoptosis was detected 48 hours after radiation using the FITC Active Caspase-3 Apoptosis Kit.

### Data Analysis and Statistics

Flow cytometry data were analyzed using FlowJo version 9.7.6. For all experiments with the exception of survival data, *p*-values were determined using Paired t-test. Survival data were plotted using Kaplan-Meier curves (GraphPad Prism v6.0a) and the *p*-value was calculated using the Mantel-Cox test

## Results

### Cold-stress suppresses radiation-induced lethality

**Fig 1:**
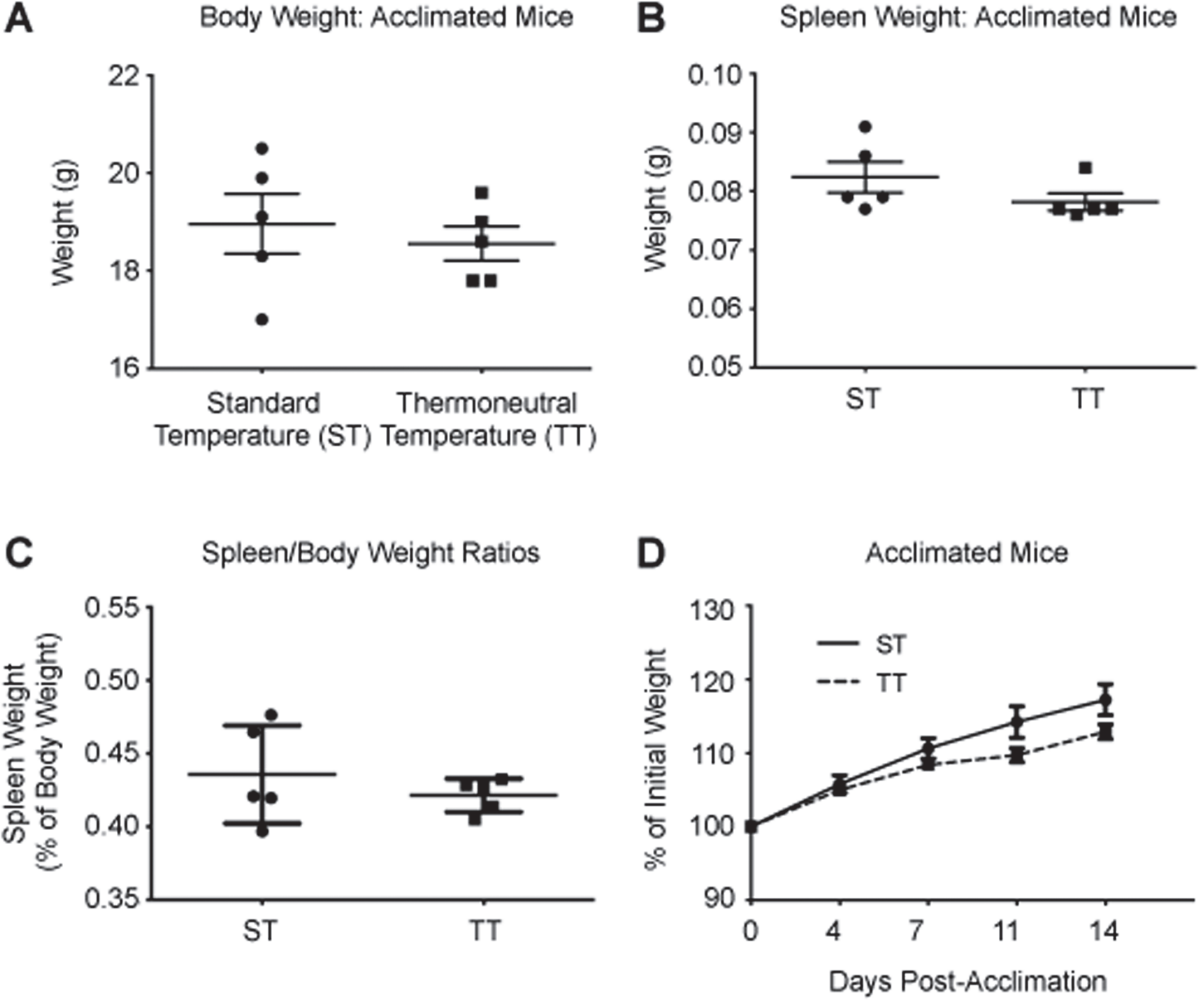
Effects of cold-stress on steady-state hematopoiesis. C57BL/6 mice were acclimated to either cold-stress (22°C, ST) or thermoneutrality (30°C, TT) for two weeks prior to all experiments as described in the Materials and Methods section. A: Average body weight; B: spleen weight; C: spleen to body weight ratio of acclimated mice. D: Average percentage of initial weight of animals during the acclimation period. For all panels, data are presented as mean ± SEM (n = 5 for all panels).

To examine the effect of mild cold-stress on hematopoiesis, we housed mice at two temperatures; the standard (IACUC-required) housing temperature (ST: 22°C) and at a thermoneutral temperature (TT: 30°C) [40]. Mice were acclimated to ST and TT conditions for 2 weeks prior to analysis of their baseline hematopoietic potential. Following acclimation, mice housed at both ST and TT maintain a similar body temperature around 37°C [23,41]. Cold-stress had no effect on body weight or spleen weight (Fig. 1A-1C) and the body weight did not change during the acclimation period (Fig. 1D).

**Fig 2:**
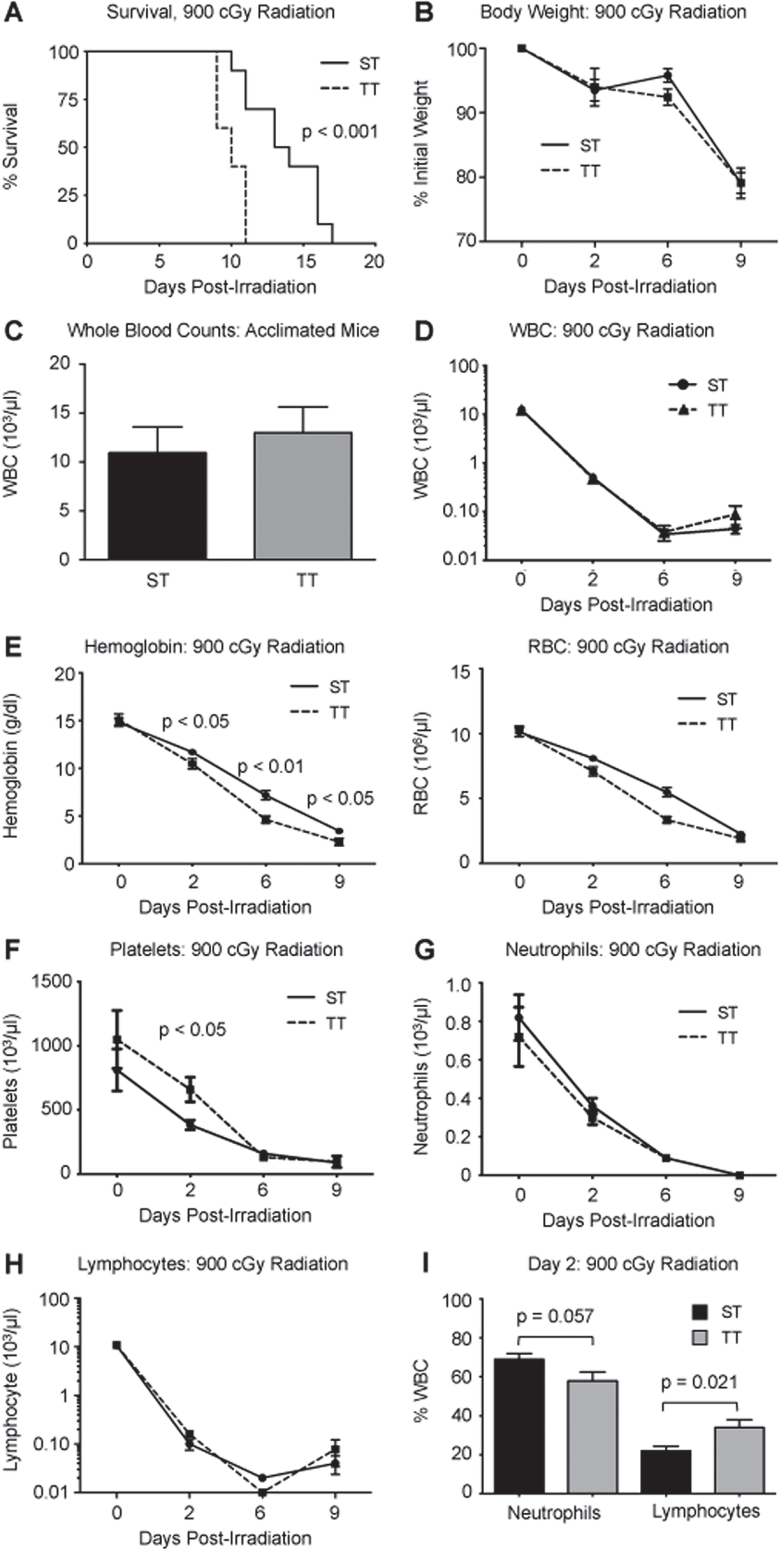
Impact of cold-stress following lethal irradiation. After acclimation, C57BL/6 mice were irradiated with 900 cGy and assessed 2, 6, and 9 days following radiation treatment. A: Kaplan-Meier curves indicating survival of mice housed at ST (solid line) *versus* TT (dotted line) following irradiation (n = 10). P-value was calculated using the Mantel-Cox test. B: Average body weight of mice housed at ST (solid line) *versus* TT (dotted line) following irradiation (n = 10). C: Average peripheral white blood cell (WBC) count (right) in acclimated mice mice. D: Average peripheral white blood cell count (WBC) of mice housed at ST (solid line, circles) *versus* TT (dotted line, triangles) following irradiation (n = 10). E-H: Average levels of hemoglobin (E), red blood cells (RBC, F), platelets (F), neutrophils (G), and lymphocytes (H) in mice housed at ST (solid line) *versus* TT (dotted line) following irradiation (n = 10 for all panels). I: Average percentage of neutrophils (left) and lymphocytes (right) in white blood cells (WBC) in ST (black bar) *versus* TT mice (gray bar, n = 10). For all panels, data are presented as mean ± SEM and p-values calculated using Student’s t test.

To determine if cold-stress affects the response to lethal hematopoietic injury, we irradiated acclimated C57BL/6 mice with a lethal dose of 900 cGy total body irradiation (TBI). Acclimation at ST significantly prolonged survival by an average of 3.5 days compared to TT mice. (Fig. 2A, p < 0.001). There was no significant difference in body weight or in total white blood cell counts between the two groups over the course of the experiment (Fig. 2A-2C). Hemoglobin levels were significantly higher in mice acclimated at ST compared to TT 2, 6, and 9 days post irradiation (Fig. 2E). A similar effect was observed on the total number of red blood cells. There was a transient increase in platelet numbers in mice acclimated at TT compared to ST 2 days post irradiation (Fig. 2F, p < 0.05). We did not observe significant changes in the total numbers of neutrophils or lymphocytes over the course of the experiment (Fig. 2G and 2H). However, we did observe a short-term trend towards an increased percentage of whole blood cells that were neutrophils in ST mice 2 days after irradiation compared to TT mice (Fig. 2I). There was also a significant decrease in the percentage of white blood cells of the lymphocyte lineage in ST *versus* TT mice (p < 0.05). These data indicate that housing mice under conditions that induce cold-stress results in increased survival following a lethal dose of radiation that is associated with increased levels of hemoglobin and red blood cells.

### Lineage-negative (Lin-), Sca-1+, c-kit+ bone marrow cells from mice under cold-stress are more resistant to radiation-induced apoptosis

**Fig 3:**
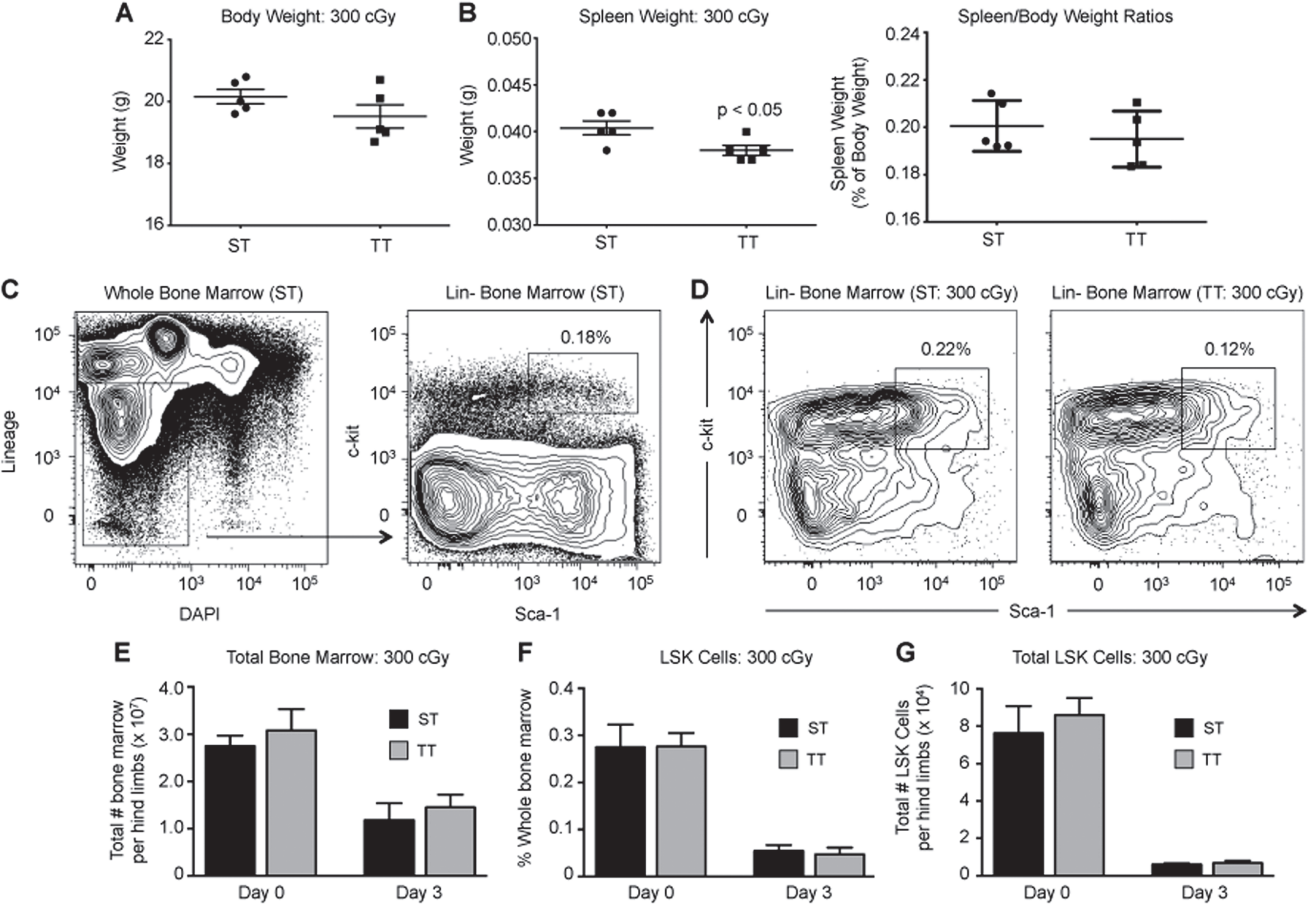
Effect of cold-stress on LSK cells following sub-lethal irradiation. Acclimated C57BL/6 mice were irradiated with 300 cGy TBI. Bone marrow cells were collected 3 days after irradiation. A-B: Average body weight (A) and spleen weight (B, left) of mice housed at ST *versus* TT 3 days following radiation (n = 5). B, right: Average ratio of spleen to body weight in acclimated mice following irradiation. C: Representative flow cytometry analysis of lineage-negative (Lin-), Sca-1+, c-kit+ (LSK cells) in bone marrow of acclimated mice. The percentage of whole bone marrow cells expressing the LSK immunophenotype is indicated. D: Representative flow cytometry analysis of LSK cells in bone marrow of sub-lethally-irradiated mice acclimated to ST (left) or TT (right) conditions. In each contour plot, the percentage of whole bone marrow cells expressing the LSK immunophenotype is indicated. E. Average number of total bone marrow cells per hind limbs harvested from ST (black bar) and TT (gray bar) mice at steady-state (Day 0) and 3 days following irradiation (n = 5). F: Average percentage of whole bone marrow cells with the LSK immunophenotype in ST (black bar) and TT (gray bar) mice at steady state and 3 days following irradiation (n = 5). G. Average number of LSK cells per hind limbs in ST (black bar) and TT (gray bar) mice at steady state and 3 days following irradiation (n = 5). Number of LSK cells was determined for each individual mouse by multiplying values in panel E by values in panel F. For all panels, data are presented as mean ± SEM and p-values calculated using Student’s t test.

While acclimation to either housing temperature resulted in similar decreases in the numbers of mature blood cells, the mice under cold-stress still exhibited increased survival. One possible explanation is that cold-stress resulted in increased numbers of HSPCs following TBI. In order to detect significant numbers of HSPCs cells, we tested this by irradiating ST and TT mice with a sub-lethal dose of 300 cGy TBI and analyzed the bone marrow 3 days post-irradiation. At this dose, there were no significant changes in body weight between the experimental groups although spleen weight was significantly reduced in TT mice (Fig. 3A and 3B). We compared hematopoietic stem and progenitor cell (HSPC) populations in the bone marrow from unirradiated and irradiated ST and TT mice using flow cytometry to detect a broadly defined HSPC population using lineage-negative (Lin-), Sca-1+, c-kit+ immunophenotype (LSK) (Fig. 3C and 3D). The LSK population is a heterogenous mix of long-term hematopoietic stem cells (HSCs), short-term HSCs, and multipotent progenitor cells [42,43]. There was no difference in the total number of whole bone marrow cells harvested from both hind limbs or in the percentage of whole bone marrow cells with the LSK phenotype in unirradiated and irradiated mice acclimated to either housing temperature (Fig. 3E and 3F). We also calculated the total number of LSK cells per hind limbs and found no detectable difference between the experimental groups (Fig. 3G). Thus, these data indicate that mild cold-stress does not result in significant perturbation of LSK numbers.

**Fig 4:**
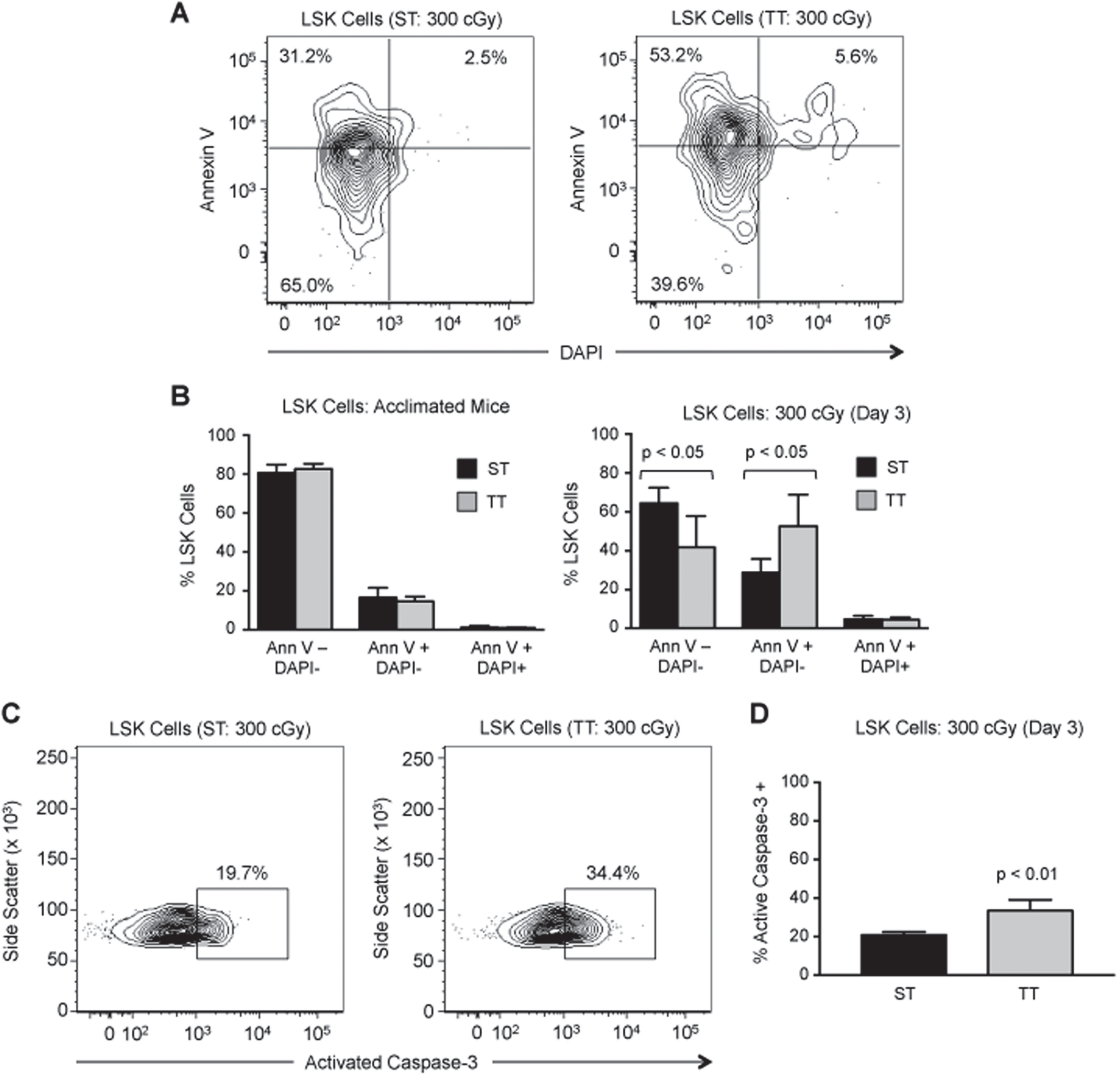
Effect of cold-stress on apoptosis following sub-lethal irradiation. A: Representative flow cytometry analysis of apoptosis in LSK bone marrow cells in ST (left) *versus* TT mice (right) 3 days following 300 cGy sub-lethal TBI. Detection of apoptosis was performed by staining cells with Annexin V and DAPI as described under Materials and Methods. Percentages of live (Annexin V-/DAPI-), actively apoptotic (Annexin V+/DAPI-), and dead (Annexin V+/DAPI+) LSK cells are indicated in each contour plot. B: Average percentage of live (Annexin V-/DAPI-), (Annexin V+/DAPI-), and dead (Annexin V+/DAPI+) LSK cells in ST (black bar) and TT (gray bar) mice at steady-state (left) and 3 days after sub-lethal irradiation (right; n = 5). C: Representative flow cytometry analysis of apoptosis (as defined by activation of caspase-3) in LSK bone marrow cells in ST (left) *versus* TT mice (right) 3 days following 300 cGy sub-lethal TBI. D: Average percentage of active caspase-3+ LSK cells in ST *versus* TT mice 3 days after sub-lethal irradiation (right; n = 5). For all panels, data are presented as mean ± SEM and p-values calculated using Student’s t test.

However, it is possible that cold-stress affects LSK cell proliferation or survival. To test this, we analyzed bone marrow cells from ST and TT mice 3 days after sub-lethal radiation. We quantified the percentage of LSK cells undergoing apoptosis by staining for Annexin V and DAPI; LSK cells actively undergoing apoptosis were defined as Annexin V+/DAPI- (Fig. 4A). There was no difference in basal apoptosis in LSK cells between ST and TT mice (Fig. 4B). However, following sub-lethal irradiation, we observed a significant 1.7-fold increase in the percentage of apoptotic LSK cells in TT mice compared to ST mice (p < 0.05). These data were confirmed through staining bone marrow cells for activated caspase-3, an alternative marker for apoptosis (p < 0.01, Fig. 4C and 4D). Together, these data indicate that cold-stress offers protection to LSK cells from pro-apoptotic effects of radiation. There was no difference in cell cycle distribution of LSK cells between ST and TT mice after irradiation (S1 Fig.).

### Cold-stress regulates HSPC apoptosis through β-adrenergic signaling

**Fig 5:**
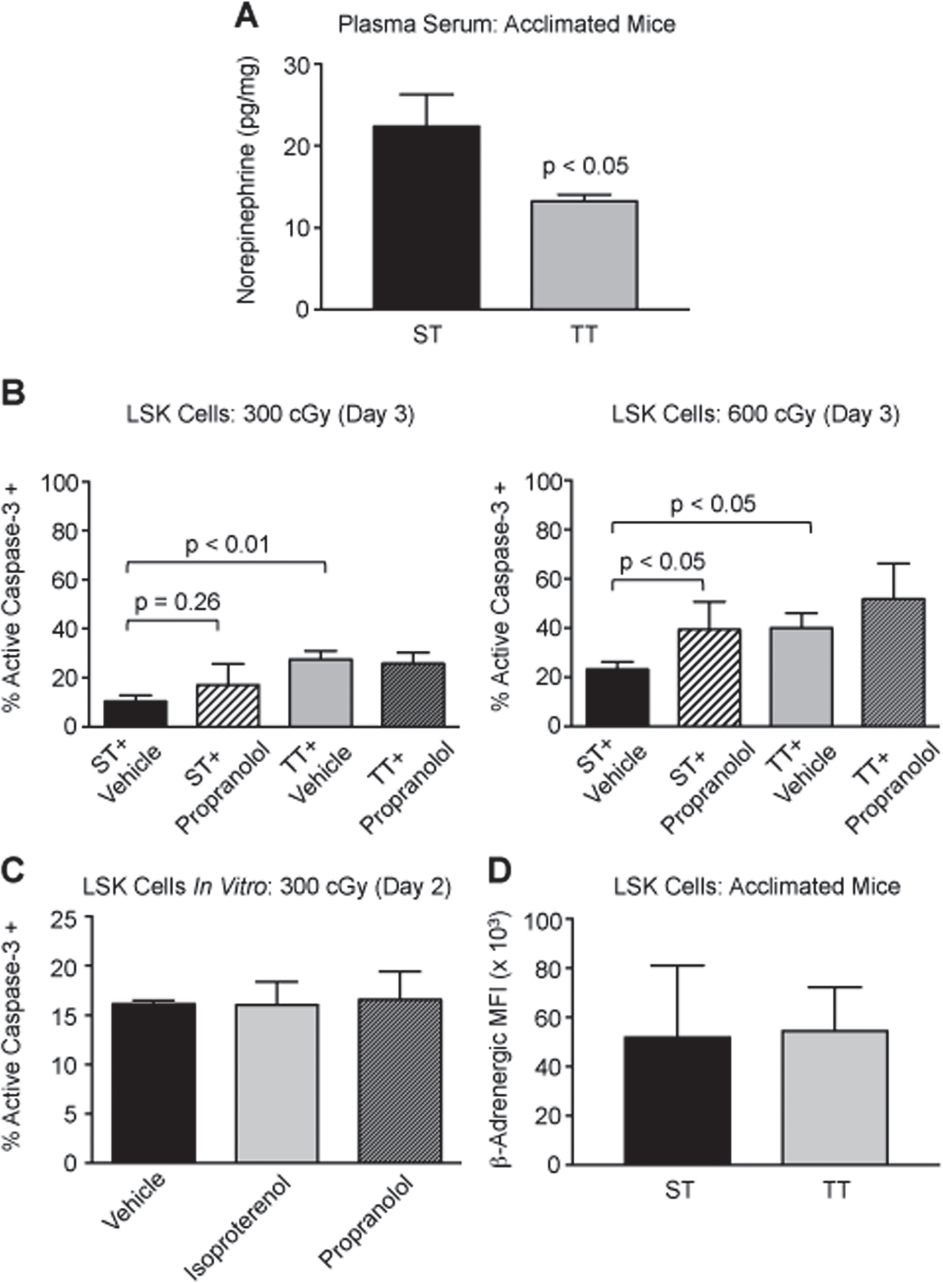
Effect of β-adrenergic inhibition on the response to cold-stress. A: Average concentration of norepinephrine in plasma serum (measured by ELISA) in from ST (black bar) and TT (gray bar) mice (n = 5). B: Average percentage of active caspase-3+ LSK cells in ST mice treated with saline vehicle or propranolol *versus* TT mice treated with saline vehicle or propranolol 3 days after 300 cGy (left) or 600 cGy (right) TBI (n = 5). C: Percentage of active caspase-3+ LSK cells 2 days after irradiation with 300 cGy *in vitro* (n = 2). Cells were cultured for 48 hours with vehicle isoproterenol, or propranolol prior to irradiation. D: Average MFI of β2-adrenergic receptor on LSK cells from ST and TT mice (n = 2). For panels A-D, data are presented as mean ± SEM and p-values calculated using Student’s t test.

Mice adapt to decreased ambient temperature through increased signaling *via* the sympathetic nervous system[44–46]. Since signaling through the β-adrenergic receptor can protect cells from apoptosis, we tested the hypothesis that cold-stress-mediated inhibition of HSPC apoptosis was regulated through the β-adrenergic signaling pathway[47]. Norepinephrine levels were 1.7-fold higher in the plasma serum of ST mice compared to TT mice (p < 0.05, Fig. 5A). To test the functional consequences of inhibiting β-adrenergic signaling, we injected acclimated ST and TT mice with 10 mg/kg propanolol (a non-selective beta-blocker) every day for 3 days starting immediately after sub-lethal irradiation. As expected, we detected a significant decrease in the percentage of active caspase-3+ LSK cells following 300 cGy doses of radiation in ST mice compared to TT mice (Fig. 5B). However, treating ST mice with propanolol partially abrogated the protective effects of cold-stress on HSPCs following radiation. This effect was even more pronounced when a higher dose of radiation (600 cGy) was used. Propranolol had no effect on apoptosis of LSK cells in TT mice at either dose. These data suggest that cold-stress protects LSK cells from radiation through β-adrenergic signaling.

To determine if the effects of β-adrenergic signaling were directly protecting HSPCs from apoptosis following radiation, we sorted LSK cells from ST mice. We cultured the cells at 37°C for two days in serum-free media supplemented with cytokines and then treated with either propranolol or with the β-adrenergic agonist isoproterenol. After two days of treatment we irradiated the cells with 300 cGy. We were unable to observe a difference in the percentage of apoptotic cells with co-treatment of either propranolol or isoproterenol (Fig. 5C). This was not due to deficiency of β-adrenergic receptor expression as LSK cells from ST and TT mice exhibit detectable levels of the β-adrenergic receptor (Fig. 5D). Thus, these data suggest that increased β-adrenergic signaling in cold-stressed mice is indirectly protecting LSK cells from radiation-induced apoptosis.

## Discussion

The behavioral preference of mice to spend a majority of time at thermoneutrality, thus reducing as much as possible the metabolic effort needed for heat production, has been well established [1,27,48]. Decades of research have demonstrated that standard housing temperature for laboratory mice results in mild chronic cold-stress even though Institutional Animal Care and Use Committees mandate these housing conditions. These studies have also demonstrated that mice adapt to cold-stress and are able to maintain a normal body temperature despite significant physiologic changes, including increased heart rate and blood pressure. In this study, we demonstrated that acclimatization to cold-stress prolonged survival from lethal TBI and inhibited apoptosis of hematopoietic stem and progenitor cells in the bone marrow. We did not observe any gross changes in steady-state hematopoiesis in mice under cold-stress compared to thermal neutrality, an observation consistent with previous studies demonstrating that some effects of cold-stress on animal physiology are not apparent until the introduction of an additional stress, such as a tumor[22,23,29].

Cold-stress activates the sympathetic nervous system to initiate a norepinephrine-driven stress signaling response suggesting a potential mechanism for our observations[46]. Our data are supported by studies over the past decades on the effects of beta-adrenergic signaling on hematopoiesis in conditions besides cold stress. Induction of b-adrenergic signaling induces DNA synthesis in HSPCs [49]. Circadian rhythms utilize β-adrenergic signaling to regulate peripheral mobilization of HSPCs[50,51]. Increased β-adrenergic signaling under conditions of social stress has been observed to enhance increased myelopoiesis[52].

Our observation that inhibition of β-adrenergic signaling in cold-stressed mice reversed the anti-apoptotic effect following radiation indicates that this pathway contributes significantly to cytoprotection of HSPCs. This observation is in agreement with our observation that β2-adrenergic pathway regulated chemoresistance in murine tumor models [53]. Previous studies have demonstrated a similar result that signaling through the β-adrenergic receptor can protect cells from apoptosis[47,54,55]. Treatment of mice with the β2-adrenergic agonist albuterol enhanced erythropoiesis following treatment with busulfan, a chemotherapy agent that is toxic to HSPCs[56]. Similarly, administration of norepinephrine promoted survival of myeloid progenitors in mice following a lethal dose of carboplatin[57].

The molecular mechanisms by which cold-stress can regulate apoptosis of HSPCs are not clear. The anti-apoptotic effects of β-adrenergic signaling may be mediated in part through Bad, a pro-apoptotic molecule that is activated by adrenergic stress and inactivated by administration of a β-adrenergic antagonist[54]. Another potential target is Bcl-2, which is upregulated by adrenergic stress[55,58]. Given that cold-stress affects multiple pathways in addition to β-adrenergic signaling, it is possible that other molecular mechanisms may contribute, including heat shock proteins and pro-survival pathways involved in metabolic regulation such as Akt/mTor.

While our studies have shown that housing at sub-thermoneutrality results in reduced apoptosis following TBI, other studies in which body temperature is actually elevated (*i*.*e*., hyperthermia) have been done by our group and others. Induction of whole-body hyperthermia immediately prior to TBI resulted in increased numbers of leukocytes in the bone marrow[59,60]. We have observed a similar effect in which neutrophil and HSPC numbers are increased when TBI is followed by a transient increase in body temperature[61]. It is important to note that the work we have presented here is distinct from other studies in which body temperature is raised in experimental animals. In this work, we compared cold-stressed animals to those acclimated to thermoneutral temperatures without affecting body temperature.

Our analysis of the HSPC population was based on the LSK immunophenotype. This population is heterogeneous and harbors different types of progenitors, including long- and short-term HSCs and multipotent progenitors[43]. Thus, it is formally possible that cold-stress preferentially affects one of these populations. Further work using functional assays is still required to fully elucidate the effects of cold-stress on specific stem and progenitor cell populations.

Our data do not clearly demonstrate whether the effects of norepinephrine on HSPCs following radiation are direct or indirect. We observed that HSPCs expressed β-adrenergic receptors, in agreement with other studies of mice and human HSPCs [62–64]. However, the data indicating that treatment with a β-adrenergic agonist was insufficient to protect cells *in vitro* suggests a model in which cold-stress mediated β-adrenergic signaling suppresses apoptosis through indirect mechanisms. This model is supported by data indicating that norepinephrine regulates HSPC mobilization via the osteoblast niche cells[51,65]. This indirect regulation of the bone marrow microenvironment, through osteoblasts or other components, may also protect HSPCs from radiation. It should also be noted that our data do not exclude a role for b-adrenergic signaling pathways[57].

Together, our data support a model in which activation of β-adrenergic signaling protects hematopoietic stem and progenitor cells from apoptotic stresses such as radiation. While the mechanisms by which β-adrenergic signaling mediates protection from radiation induced apoptosis remain to be elucidated, this work suggests that pharmacologic induction of β-adrenergic signaling either through isoproterenol, which acts through both β1- and β2-adrenergic receptors, or a β2 specific agonist may be an effective countermeasure for patients with acute radiation. Further work will be required to elucidate the ability of adrenergic stimulation to protect cells from apoptotic stimuli.

## Supporting Information

S1 Fig Cell cycle status of LSK cells in cold-stressed mice following sub-lethal irradiation. Average percentage of LSK cells in each cell cycle phase in ST (black bar) *versus* TT (gray bar) mice 3 days after sub-lethal irradiation (n = 5).
(TIF)
