# Supplementary figures and images for: Standard Sub-Thermoneutral Caging Temperature Influences Radiosensitivity of Hematopoietic Stem and Progenitor Cells
Source: PLoS One. 2015 Mar 20;10(3):e0120078. doi: 10.1371/journal.pone.0120078 (PMC4368554; doi:10.1371/journal.pone.0120078)

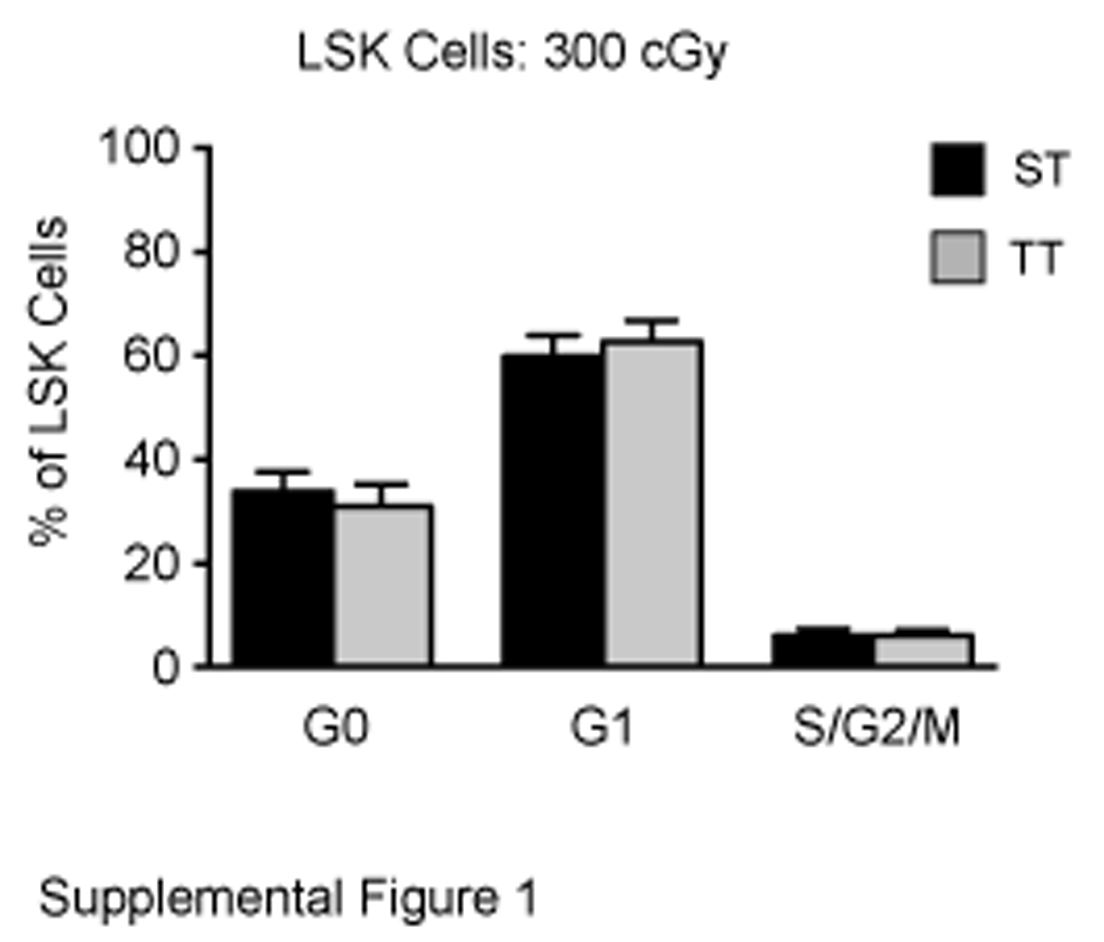

Supplement: S1 Fig — Average percentage of LSK cells in each cell cycle phase in ST (black bar) versus TT (gray bar) mice 3 days after sub-lethal irradiation (n = 5). (TIF) [file pone.0120078.s001.tif]
